# The association between anxiety disorders and in‐hospital outcomes in patients with myocardial infarction

**DOI:** 10.1002/clc.23358

**Published:** 2020-03-18

**Authors:** Pengyang Li, Xiaojia Lu, Mark Kranis, Fangcheng Wu, Catherine Teng, Peng Cai, Zeba Hashmath, Bin Wang

**Affiliations:** ^1^ Department of Medicine Saint Vincent Hospital Worcester Massachusetts USA; ^2^ Department of Cardiology the First Affiliated Hospital of Shantou University Medical College Shantou Guangdong China; ^3^ Department of Cardiology Saint Vincent Hospital Worcester Massachusetts USA; ^4^ Department of Medicine Memorial Hospital West Pembroke Pines Florida USA; ^5^ Department of Medicine, Greenwich Hospital Yale New Haven Health Greenwich Connecticut USA; ^6^ Department of Mathematical Sciences Worcester Polytechnic Institute Worcester Massachusetts USA

**Keywords:** anxiety disorders, in‐hospital outcomes, myocardial infarction

## Abstract

**Background:**

Anxiety disorders are prevalent in patients with myocardial infarction (MI), but the effects of anxiety disorders on in‐hospital outcomes within MI patients have not been well studied.

**Hypothesis:**

To examine the effects of concurrent anxiety disorders on in‐hospital outcomes in MI patients.

**Methods:**

We conducted a retrospective cohort study in patients with a principal diagnosis of MI with and without anxiety disorders in the National Inpatient Sample 2016. A total of 129 305 primary hospitalizations for acute MI, 35 237 with ST‐segment elevation myocardial infarction (STEMI), and 94 068 with non‐ST elevation myocardial infarction (NSTEMI) were identified. Of these, 13 112 (10.1%) had anxiety (7.9% in STEMI and 11.0% in NSTEMI). We compared outcomes of anxiety and nonanxiety groups after propensity score matching for the patient and hospital demographics and relevant comorbidities.

**Results:**

After propensity score matching, the anxiety group had a lower incidence of in‐hospital mortality (3.0% vs 4.4%, *P* < .001), cardiac arrest (2.1% vs 2.8%, *P* < .001), cardiogenic shock (4.9% vs 5.6%, *P* = .007), and ventricular arrhythmia (6.7% vs 7.9%, *P* < .001) than the nonanxiety group. In the NSTEMI subgroup, the anxiety group had significantly lower rates of in‐hospital mortality (2.3% vs 3.5%, *P* < .001), cardiac arrest (1.1% vs 1.5%, *P* = .008), and cardiogenic shock (2.8% vs 3.5%, *P* = .008). In the STEMI subgroup, we found no differences in in‐hospital outcomes (all *P* > .05) between the matched groups.

**Conclusion:**

Although we found that anxiety was associated with better in‐hospital outcomes, subgroup analysis revealed that this only applied to patients admitted for NSTEMI instead of STEMI.

## 1 INTRODUCTION

Myocardial infarction (MI) is characterized by chest pain and/or dyspnea as a result of myocardial ischemia and injury. MI can be divided into ST‐segment elevation myocardial infarction (STEMI) and non‐ST elevation myocardial infarction (NSTEMI) based on electrocardiography changes. With the increasing utilization of medical therapies and reperfusion interventions, the short‐term mortality of MI has been steadily decreasing over the past decade. In‐hospital mortality is 5.5% among STEMI and 3.9% among NSTEMI.^1^ Many preexisting factors have been identified that affect the outcomes of MI, such as age, sex, prior MI,^2^ peripheral artery disease (PAD),^3^ chronic obstructive pulmonary disease (COPD),^4^ obstructive sleep apnea (OSA),^5^ chronic kidney disease (CKD),^6^ prior stroke,^7^ and traditional cardiovascular risk factors (hypertension, smoking, dyslipidemia, and diabetes).^8^ In recent years, numerous studies have shown that psychosocial factors, especially depression disorders, play an adverse role in the prognosis for MI and maybe even more significant than traditional cardiovascular risk factors.^9^, ^10^, ^11^ Although highly overlapped with depression disorders, anxiety disorders as a cardiovascular risk factor have not been studied as extensively as other psychosocial factors. Anxiety disorders are prevalent in patients with MI,^12^ and the effects of them can manifest pre‐ or post‐MI. Previous studies have shown that preexisting anxiety is an independent predictor of MI,^13^, ^14^ and post‐MI anxiety will worsen the long‐term prognosis of MI, associated with increased medical consumption, a higher incidence of cardiovascular events, and the greater out of hospital mortality.^15^, ^16^, ^17^ Some studies focused on the effect of preexisting anxiety on the long‐term prognosis of post‐MI. For instance, Smeijers et al found that preexisting anxiety will increase the 10‐year mortality risk among MI patients.^18^ However, a few studies that focus on the short‐term outcomes for patients with preexisting anxiety at the time of AMI, especially in‐hospital outcomes which contributes the most of mortality in MI patients. Interestingly, a recent study by Pino et al demonstrated that patients with anxiety and/or depression at the time of STEMI paradoxically have a better in‐hospital outcome.^19^ However, the study did not distinguish between anxiety and depression disorders, and only STEMI patients were studied. Our study aims to investigate the impact of preexisting anxiety disorders on in‐hospital outcomes in both NSTEMI and STEMI patients after adjusting for other well‐established risk factors and depressive disorders.

## 2 METHOD

We conducted a retrospective cohort study using the National Inpatient Sample (NIS) 2016 database. The NIS is part of the Healthcare Cost and Utilization Project (HCUP) and includes administrative and demographic data involving a stratified sample of 20% (more than 7 million each year) of inpatient hospitalizations in the United States, excluding rehabilitation and long‐term care facilities.^20^ It contains the following variables: primary and secondary diagnoses, patient demographic characteristics, hospital characteristics, total charges, expected payer, discharge status, length of stay (LOS), and severity and comorbidity measures. Because of the large sample size, the NIS has been used in developing national and regional estimates. All the discharge diagnoses in the 2016 NIS database are identified by the International Classification of Diseases, Tenth Revision, Clinical Modification (ICD‐10‐CM) Codes.

**Figure 1:**
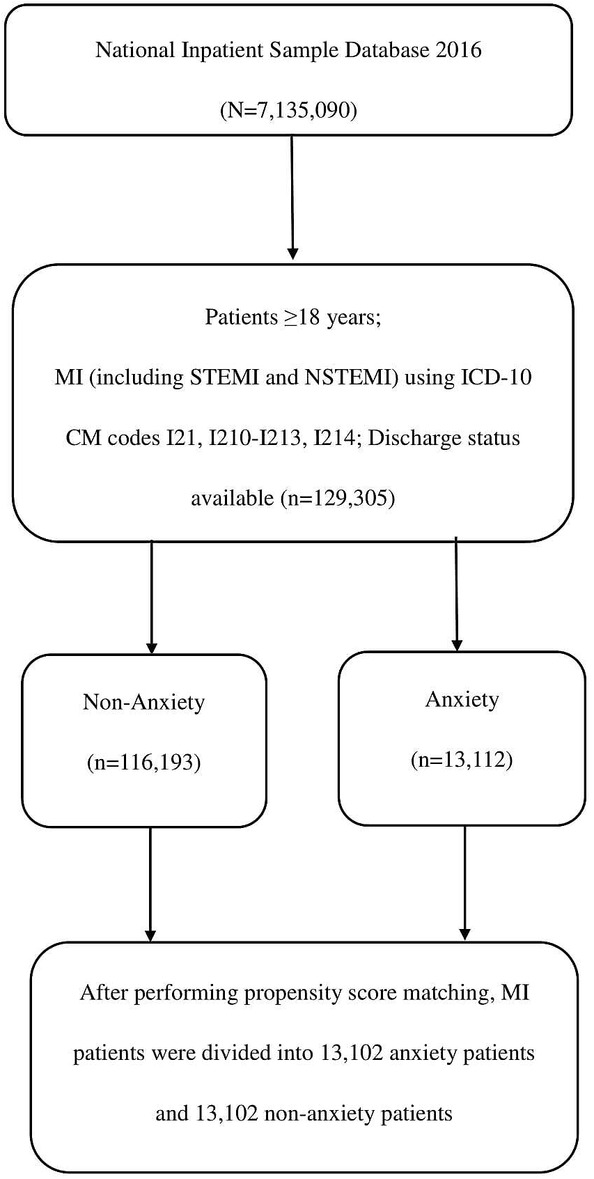
Flow chart of the selection process for the final patient sample used in this study. Inclusion criteria were applied to the National Inpatient Sample 2016 database. All eligible patients were matched 1:1 based on propensity scoring to generate the anxiety vs nonanxiety comparison cohorts. ICD‐10‐CM code, Tenth Revision, Clinical Modification Code. MI, myocardial infarction; NSTEMI, non‐ST elevation myocardial infarction; STEMI, ST‐segment elevation myocardial infarction

MI patients over 18 years old who were admitted to hospital from 1 January 2016 to 31 December 2016 were selected by the ICD‐10‐CM codes. Patients with missing information on discharge status were excluded. Eligible patients with MI were further grouped based on whether they had anxiety disorders or not. The selection process for the final patient sample used in this study was illustrated in Figure 1.

Review by an institutional review board is not required for use of NIS.

## 3 VARIABLES

The baseline data included age, sex, race, geographic location, household income, primary payer, hospital‐level characteristics, such as hospital type, region and bed size, and risk factors for adverse outcomes of MI: smoking, hypertension, hyperlipidemia, diabetes mellitus (DM), obesity, history of MI, OSA, CKD, history of stroke, PAD, COPD, and depression disorders. All the comorbidities mentioned above were extracted directly from the NIS database by the ICD‐10‐CM codes (Table S1).

## 4 OUTCOMES

The primary outcomes were defined as in‐hospital death, cardiac arrest, and cardiogenic shock. The secondary outcomes were LOS, total hospitalization costs, and serious in‐hospital complications: ventricular arrhythmia (VA), acute congestive heart failure (CHF), acute kidney injury (AKI), and acute respiratory failure (ARF). All the ICD‐10 codes representing the outcomes were shown in Table S1.

## 5 STATISTICAL ANALYSES

Categorical variables are represented by numbers and proportions by chi‐square test. Continuous variables are presented by mean and standard deviation (SD). A propensity score‐matched analysis of the anxiety vs the nonanxiety group was performed to reduce the selection bias within the unmatched cohort.

To adjust for age, gender, race, mean household income, hospital type, hospital region, and risk factors for adverse outcome of MI, we built a multivariate logistic regression model to measure a propensity score. We conducted a nearest neighbor matching of both groups with a caliper match tolerance of 0.25 to decide the subsequent individually matched propensity score. Next, the primary and secondary outcomes between the anxiety and without anxiety groups were compared in both the unmatched and the propensity score‐compatible cohorts. Patients with MI were further divided into STEMI and NSTEMI subgroups. A propensity score‐matched analysis of the anxiety vs nonanxiety groups was performed in the STEMI and NSTEMI subgroups, respectively. Finally, the same primary outcomes (death, cardiac arrest, and cardiogenic shock) and secondary outcomes (LOS, total cost, VA, acute CHF, AKI, and ARF) between the anxiety and without anxiety groups in the subgroup were measured. The patient selection process for the subgroups was illustrated in Figures S1 and S2. All statistical analyses were performed by the R statistics software. The value of *P* < .05 was considered significant.

## 6 RESULTS

A total of 129 305 acute MI patients (including STEMI 35 237, 27.3% and NSTEMI 94 068, 72.7%) were identified, in which 13 112 (10.1%) had anxiety disorders (2770 in STEMI and 10 342 in NSTEMI).

In the unmatched cohort of MI, compared with the patients without anxiety, the patients with anxiety tended to be younger (65.5 ± 13.7 vs 67.2 ± 13.6, *P* < .001), female (56.1% vs 36.0%, *P* < .001), white (79.7% vs 70.1%, *P* < .001), and had a higher incidence of comorbidities: smoking (29.4% vs 23.0%, *P* < .001), hypertension (56.9% vs 53.7%, *P* < .001), hyperlipidemia (68.0% vs 64.7%, *P* < .001), obesity (21.0% vs 18.4%, *P* < .001), depression (32.1% vs 5.8%, *P* < .001), history of MI (17.9% vs 15.0%, *P* < .001), OSA (11.7% vs 8.4%, *P* < .001), history of stroke (11.3% vs 10.0%, *P* < .001), PAD (13.3% vs 12.5%, *P* = .005), and COPD (26.5% vs 17.0%, *P* < .001). Interestingly, the incidence of DM (36.4% vs 38.9%, *P* < .001) and CKD (21.6% vs 23.7%, *P* < .001) was less in the group with anxiety. After propensity score matching, each group consisted of 13 102 patients, although sex and race still differ between the two groups. Like the unmatched MI, the pre‐MI anxiety group was proportionately more female (56.1% vs 54.4%, *P* = .013), and white (79.7% vs 76.6%, *P* < .001) (Table 1).

**Table 1:** Baseline characteristics of patients with MI

	Unmatched cohort			Propensity‐matched cohort		
Variables	MI without anxiety	MI with anxiety	*P* value	MI without anxiety	MI with anxiety	*P* value
n	116 193	13 112		13 102	13 102	
Age, (mean [SD])	67.2 (13.6)	65.5 (13.7)	<.001	65.5 (13.6)	65.5 (13.7)	.966
Sex, n (%)			<.001			.013
Male	74 363 (64.0)	5749 (43.8)		5975 (45.6)	5749 (43.9)	
Female	41 775 (36.0)	7356 (56.1)		7123 (54.4)	7346 (56.1)	
Unknown	55 (0.0)	7 (0.1)		4 (0.0)	7 (0.1)	
Race, n (%)			<.001			<.001
White	81 460 (70.1)	10 449 (79.7)		10 040 (76.6)	10 440 (79.7)	
Black	13 061 (11.2)	876 (6.7)		1361 (10.4)	875 (6.7)	
Hispanic	9291 (8.0)	816 (6.2)		785 (6.0)	816 (6.2)	
Asian/Pacific Islander	3215 (2.8)	139 (1.1)		190 (1.5)	139 (1.1)	
Native American	623 (0.5)	50 (0.4)		51 (0.4)	50 (0.4)	
Other	3361 (2.9)	259 (2.0)		151 (1.2)	259 (2.0)	
Unknown	5182 (4.5)	523 (4.0)		524 (4.0)	523 (4.0)	
Patient location, n (%)			<.001			.478
“Central” counties of metro areas of > = 1 million population	29 511 (25.4)	2878 (21.9)		3005 (22.9)	2878 (22.0)	
“Finge” counties of metro areas of > = 1 million population	27 020 (23.3)	3131 (23.9)		3081 (23.5)	3127 (23.9)	
Counties in metro areas of 250 000‐999 999 population	23 651 (20.4)	2700 (20.6)		2659 (20.3)	2698 (20.6)	
Counties in metro areas of 50 000‐249 999 population	11 845 (10.2)	1541 (11.8)		1467 (11.2)	1540 (11.8)	
Micropolitan counties	13 160 (11.3)	1576 (12.0)		1607 (12.3)	1575 (12.0)	
Non metropolitan or micropolitan counties	10 587 (9.1)	1256 (9.6)		1255 (9.6)	1254 (9.6)	
NA	419 (0.4)	30 (0.2)		28 (0.2)	30 (0.2)	
Mean household income, n (%)			0.01			.787
$1‐$42 999	35 151 (30.3)	4088 (31.2)		4042 (30.9)	4086 (31.2)	
$43 000‐$53 999	30 737 (26.5)	3503 (26.7)		3545 (27.1)	3499 (26.7)	
$54 000‐$70 999	27 008 (23.2)	3035 (23.1)		3072 (23.4)	3033 (23.1)	
$71 000 or more	21 112 (18.2)	2278 (17.4)		2253 (17.2)	2276 (17.4)	
Unknown	2185 (1.9)	208 (1.6)		190 (1.5)	208 (1.6)	
Primary payer, n (%)			<.001			.067
Medicare	66 386 (57.1)	7697 (58.7)		7606 (58.1)	7690 (58.7)	
Medicaid	10 513 (9.0)	1484 (11.3)		1427 (10.9)	1481 (11.3)	
Private including HMO (Health maintenance organization)	30 083 (25.9)	3133 (23.9)		3194 (24.4)	3133 (23.9)	
Self‐pay	5249 (4.5)	434 (3.3)		525 (4.0)	434 (3.3)	
No charge	495 (0.4)	37 (0.3)		43 (0.3)	37 (0.3)	
Other	3333 (2.9)	315 (2.4)		296 (2.3)	315 (2.4)	
Unknown	134 (0.1)	12 (0.1)		11 (0.1)	12 (0.1)	
Hospital type, n (%)			<.001			.403
Rural	8805 (7.6)	1185 (9.0)		1127 (8.6)	1184 (9.0)	
Urban nonteaching	32 592 (28.0)	3539 (27.0)		3591 (27.4)	3537 (27.0)	
Urban teaching	74 796 (64.4)	8388 (64.0)		8384 (64.0)	8381 (64.0)	
Hospital region, n (%)			<.001			.103
Northeast	20 697 (17.8)	2473 (18.9)		2402 (18.3)	2470 (18.9)	
Midwest	25 997 (22.4)	3267 (24.9)		3240 (24.7)	3262 (24.9)	
South	47 473 (40.9)	5285 (40.3)		5228 (39.9)	5283 (40.3)	
West	22 026 (19.0)	2087 (15.9)		2232 (17.0)	2087 (15.9)	
Hospital bed size, n (%)			.829			.901
Small	18 327 (15.8)	2095 (16.0)		2067 (15.8)	2094 (16.0)	
Medium	34 471 (29.7)	3877 (29.6)		3880 (29.6)	3873 (29.6)	
Large	63 395 (54.6)	7140 (54.5)		7155 (54.6)	7135 (54.5)	
Comorbidities, n (%)						
Smoking	26 672 (23.0)	3853 (29.4)	<.001	3756 (28.7)	3843 (29.3)	.242
Hypertension	62 371 (53.7)	7462 (56.9)	<.001	7396 (56.4)	7453 (56.9)	.485
DM	45 243 (38.9)	4768 (36.4)	<.001	4813 (36.7)	4764 (36.4)	.538
Hyperlipidemia	75 135 (64.7)	8910 (68.0)	<.001	8907 (68.0)	8901 (67.9)	.947
Obesity	21 336 (18.4)	2759 (21.0)	<.001	2686 (20.5)	2755 (21.0)	.3
Depression	6756 (5.8)	4214 (32.1)	<.001	4103 (31.3)	4204 (32.1)	.184
History of MI	17 406 (15.0)	2349 (17.9)	<.001	2293 (17.5)	2346 (17.9)	.4
OSA	9788 (8.4)	1532 (11.7)	<.001	1535 (11.7)	1526 (11.6)	.878
CKD	27 578 (23.7)	2834 (21.6)	<.001	2824 (21.6)	2834 (21.6)	.893
History of stroke	11 624 (10.0)	1484 (11.3)	<.001	1469 (11.2)	1445 (11.0)	.651
PAD	14 467 (12.5)	1745 (13.3)	.005	1800 (13.7)	1743 (13.3)	.312
COPD	19 739 (17.0)	3475 (26.5)	<.001	3409 (26.0)	3466 (26.5)	.432

Abbreviations: CKD, chronic kidney disease; COPD, chronic obstructive pulmonary disease; DM, diabetes mellitus; MI, myocardial infarction; NSTEMI, non‐ST elevation myocardial infarction; OSA, obstructive sleep apnea; PAD, peripheral artery disease; STEMI, ST‐segment elevation myocardial infarction; HMO, health maintenance organization.

In the STEMI group, 32 467 patients did not have anxiety and 2770 had anxiety disorders, while for 94 068 patients in the NSTEMI group, 83 726 did not have anxiety and 10 342 had anxiety disorders. Before propensity score matching, like the anxiety group of MI, the STEMI and NSTEMI anxiety groups had more young, female, white patients, and a higher incidence of comorbidities. However, fewer patients had DM (38.3% vs 42.2%, *P* < .001) and CKD (24.0% vs 28.0%, *P* < .001) in the NSTEMI with anxiety group vs the without anxiety group, while the DM (29.0% vs 30.6%, *P* = .084) and CKD (12.6% vs 12.7%, *P* = .995) did not differ in the patients in the STEMI group. After propensity score matching, race still maintains a significant difference in the anxiety group and nonanxiety group of the STEMI and NSTEMI respectively (all *P* < .001) (Table S2 and S3).

In contrast to previous studies, our study shows that patients with pre‐MI anxiety have a better in‐hospital outcome than those without. In the propensity‐matched group, patients with pre‐MI anxiety are associated with lower in‐hospital mortality (3.0% vs 4.4%, *P* < .001), cardiac arrest (2.1% vs 2.8%, *P* < .001), cardiogenic shock (4.9% vs 5.6%, *P* = .007) and VA (6.7% vs 7.9%, *P* < .001), but a longer LOS (4.7 ± 5.3 vs 4.4 ± 5.6, *P* = .001) (Table 2). In the group of NSTEMI, after propensity match, we found that the subgroup of pre‐MI anxiety had a lower incidence of in‐hospital death (2.3% vs 3.5%, *P* < .001), cardiac arrest (1.1% vs 1.5%, *P* = .008), cardiac shock (2.8% vs 3.5%, *P* = .008), and AKI (18.1% vs 19.7%, *P* = .004) (Table 3). We found no differences, however, in the all primary and secondary outcomes in the STEMI subgroup after propensity score matching, except a longer LOS in the anxiety group (4.7 ± 7.3 vs 4.1 ± 5.4, *P* = .002) (Table 4).

**Table 2:** In‐hospital outcomes of MI

	Unmatched cohort	Propensity‐matched cohort				
Variables	MI without anxiety	MI with anxiety	*P* value	MI without anxiety	MI with anxiety	*P* value
n	116 193	13 112		13 102	13 102	
Outcomes						
Death, n (%)	5687 (4.9)	391 (3.0)	<.001	571 (4.4)	391 (3.0)	<.001
Cardiac arrest, n (%)	3517 (3.0)	269 (2.1)	<.001	372 (2.8)	269 (2.1)	<.001
Cardiogenic shock, n (%)	7190 (6.2)	638 (4.9)	<.001	736 (5.6)	638 (4.9)	.007
VA, n (%)	9833 (8.5)	882 (6.7)	<.001	1041 (7.9)	881 (6.7)	<.001
Acute CHF, n (%)	22 112 (19.0)	2526 (19.3)	.525	2457 (18.8)	2523 (19.3)	.306
AKI, n (%)	22 204 (19.1)	2299 (17.5)	<.001	2282 (17.4)	2298 (17.5)	.807
ARF, n (%)	12 908 (11.1)	1609 (12.3)	<.001	1514 (11.6)	1607 (12.3)	.079
LOS, (mean [SD])	4.4 (5.5)	4.7 (5.3)	<.001	4.4 (5.6)	4.7 (5.3)	.001
Total cost, (mean [SD])	21 438.6 (23 146.7)	20 760.3 (22 320.8)	.002	20 695.9 (22 825.6)	20 763.3 (22 327.1)	.811

Abbreviations: AKI, acute kidney injury; ARF, acute respiratory failure; CHF, congestive heart failure; LOS, length of stay; MI, myocardial infarction; SD, standard deviation.

**Table 3:** In‐hospital outcomes of NSTEMI

	Unmatched cohort	Propensity‐matched cohort				
Variables	NSTEMI without anxiety	NSTEMI with anxiety	*P* value	NSTEMI without anxiety	NSTEMI with anxiety	*P* value
n	83 726	10 342		10 340	10 340	
Outcomes						
Death, n (%)	3010 (3.6)	235 (2.3)	<.001	367 (3.5)	235 (2.3)	<.001
Cardiac arrest, n (%)	1485 (1.8)	111 (1.1)	<.001	155 (1.5)	111 (1.1)	.008
Cardiogenic shock, n (%)	2974 (3.6)	292 (2.8)	<.001	360 (3.5)	292 (2.8)	.008
VA, n (%)	4441 (5.3)	452 (4.4)	<.001	503 (4.9)	452 (4.4)	.098
Acute CHF, n (%)	17 804 (21.3)	2107 (20.4)	.037	2206 (21.3)	2107 (20.4)	.093
AKI, n (%)	17 102 (20.4)	1869 (18.1)	<.001	2034 (19.7)	1869 (18.1)	.004
ARF, n (%)	8951 (10.7)	1245 (12.0)	<.001	1250 (12.1)	1245 (12.0)	.932
LOS, (mean [SD])	4.6 (5.4)	4.7 (4.6)	.201	4.6 (5.2)	4.7 (4.6)	.431
Total cost, (mean [SD])	19 922.2 (21 528.5)	19 348.8 (18 256.4)	.01	19 550.9 (19 836.5)	19 350.6 (18 257.8)	.455

Abbreviations: AKI, acute kidney injury; ARF, acute respiratory failure; CHF, congestive heart failure; LOS, length of stay; NSTEMI, non‐ST elevation myocardial infarction; SD, standard deviation.

**Table 4:** In‐hospital outcomes of STEMI

	Unmatched cohort	Propensity‐matched cohort				
Variables	STEMI without anxiety	STEMI with anxiety	*P* value	STEMI without anxiety	STEMI with anxiety	*P* value
n	32 467	2770		2768	2768	
Outcomes						
Death, n (%)	2677 (8.2)	156 (5.6)	<.001	191 (6.9)	156 (5.6)	.059
Cardiac arrest, n (%)	2032 (6.3)	158 (5.7)	.263	155 (5.6)	158 (5.7)	.907
Cardiogenic shock, n (%)	4216 (13.0)	346 (12.5)	.475	320 (11.6)	346 (12.5)	.302
VA, n (%)	5392 (16.6)	430 (15.5)	.148	419 (15.1)	430 (15.5)	0.709
Acute CHF, n (%)	4308 (13.3)	419 (15.1)	.006	376 (13.6)	419 (15.1)	.107
AKI, n (%)	5102 (15.7)	430 (15.5)	.812	439 (15.9)	430 (15.5)	.768
ARF, n (%)	3957 (12.2)	364 (13.1)	.151	323 (11.7)	364 (13.2)	.103
LOS (mean [SD])	4.1 (5.5)	4.7 (7.3)	<.001	4.1 (5.4)	4.7 (7.3)	.002
Total cost (mean [SD])	25 318.6 (26 454.5)	25 996.5 (32 795.6)	.208	25 065.3 (26 233.7)	25 998.9 (32 807.4)	.245

Abbreviations: AKI, acute kidney injury; ARF, acute respiratory failure; CHF, congestive heart failure; LOS, length of stay; SD, standard deviation; STEMI, ST‐segment elevation myocardial infarction.

## 7 DISCUSSION

To our knowledge, this study is the first to investigate the effect of preexisting anxiety disorders on in‐patient outcomes of subgroups of MI patients. Unlike previous studies, by using the national representative hospitalization database, we primarily observed that preexisting anxiety was associated with decreased in‐hospital mortality, a lower incidence of cardiac arrest, cardiogenic shock, and VA in MI inpatients. Interestingly, the same effects in mortality, cardiac arrest, and cardiogenic shock were found in patients with anxiety in the NSTEMI subgroup but not in STEMI subgroup.

It is well established that preexisting anxiety is associated with worse long‐term outcomes in MI patients.^15^, ^16^, ^17^ Several hypotheses have been proposed to explain the negative impacts of preexisting anxiety disorders on the long‐term outcomes in patients with MI, such as the change of biological behavior: heavy drinking,^21^ nicotine dependence,^22^ and lack of exercise.^23^ Another hypothesis is that anxiety can escalate the inflammatory response by activating inflammatory markers such as C‐reactive protein, interleukin‐6, and homocysteine,^24^, ^25^ which may in turn lead to the formation of the coronary thrombosis. In addition, studies showed that anxiety can interrupt the balance of autonomic nervous system by reducing the vagal tone, making a higher incidence of ventricular fibrillation and tachycardia.^26^, ^27^

Mood disorders including anxiety and depression mainly affect the body by activating the sympathetic nervous system and result in a high level of catecholamine.^28^ Fioranelli et al found that a high level of catecholamine increases the cortisol, which also leads to the increased risk of ischemic heart diseases.^29^ Interestingly, other studies suggested that catecholamine may have a protective effect of MI in acute setting. Abete et al found that elevated levels of norepinephrine in mice can reduce the area of myocardial damage during ischemia‐reperfusion.^30^ This phenomenon might be explained by the ischemic preconditioning (IPC), which is an adaptational response of brief episodes of ischemia where reperfusion serves to protect against subsequent prolonged ischemia‐reperfusion injury. Although the mechanism of IPC remains unclear, several studies have shown that the protective effects are mediated by the elevation of plasma catecholamine during brief episodes of ischemia and reperfusion.^31^, ^32^ Anxiety disorders can similarly lead to increased plasma catecholamine and can possibly mimic the process of myocardial preconditioning, which resulted in less ischemia and reperfusion injury in acute MI.

Moreover, anxiety disorders and cardiovascular diseases present with similar symptoms such as chest pain, palpitation, and dyspnea. Patients with anxiety might seek medical attention promptly and more frequently, which might lead to early diagnosis and treatment compared to patients without a preexisting diagnosis of anxiety.

Preexisting anxiety was associated with better in‐hospital outcomes in NSTEMI but not in STEMI. By definition, STEMI is a transmural myocardial infarction (full‐thickness myocardial necrosis) and is most often caused by complete and persistent occlusion of a coronary artery by thrombus, while NSTEMI is a non‐full‐wall myocardial necrosis and usually due to a decrease in blood supply via partial occlusion of the affected coronary artery.^33^ Traditionally, patients with STEMI have a substantially higher in‐hospital mortality than those with NSTEMI.^1^ The protective effects of anxiety disorders in STEMI could be less significant than in NSTEMI because the more severe myocardial necrosis in STEMI, the less involvement there is of reperfusion injury (which might be alleviated by preexisting anxiety disorders) given the underlying etiology of complete occlusion of the coronary artery in STEMI.

Our study has several limitations. First, because of its retrospective observational design, our study can help establish associations, but not causality. Further mechanistic study is warranted to verify these findings. Second, the absence of data on whether patients received antianxiety therapy in the data set prevented us from examining antianxiety therapy‐related effects. Third, we were unable to analyze the effects of other cardiovascular drug treatments, especially the β‐adrenergic receptor blockers, which antagonize the sympathetic nervous system. Fourth, a previous study showed that the sensitivity and specificity of the ICD‐10‐CM code for identifying MI was about 61.5% and 99.4%, respectively.^34^ Misclassification may be a source of bias. Finally, because of the limitations of the database, we were unable to include other possible residual confounding factors, such as access to healthcare, diet/lifestyle, and detailed socioeconomic information (which were not available in NIS and may be potential explanations for the better outcomes in NSTEMI), into our analysis.

## 8 CONCLUSIONS

In summary, although previous studies have found that anxiety is associated with a negative effect on the outcomes of MI, we found that preexisting anxiety was associated with positive short‐term prognosis of MI patients in terms of decreased in‐hospital mortality, cardiac arrest, cardiogenic shock, and VA. On the other hand, anxiety has different effects on the subtypes of MI. Preexisting anxiety plays a beneficial role in the short‐term prognosis of patients with NSTEMI with a lower incidence of in‐hospital mortality, cardiac arrest, and cardiogenic shock, but no material effects on inpatient outcomes for the patients in the STEMI group.

## CONFLICT OF INTEREST

The authors declare no potential conflict of interest.

## Supporting information


**Figure S1**. Flow chart of the selection process for the final patient sample in the STEMI subgroup used in this study. Inclusion criteria were applied to the National Inpatient Sample 2016 database. All eligible patients were matched 1:1 based on propensity scoring to generate the anxiety vs nonanxiety comparison cohorts. ICD‐10‐CM code: Tenth Revision, Clinical Modification Code. STEMI: ST‐segment elevation myocardial infarction.

**Figure S2**. Flow chart of the selection process for the final patient sample in the NSTEMI subgroup used in this study. Inclusion criteria were applied to the National Inpatient Sample 2016 database. All eligible patients were matched 1:1 based on propensity scoring to generate the anxiety vs nonanxiety comparison cohorts. ICD‐10‐CM code: Tenth Revision, Clinical Modification Code. NSTEMI: non‐ST elevation myocardial infarction.

**Table S1** International Classification of Disease, Version 10 (ICD‐10) Codes Used for Co‐Morbidity and Outcomes Identification

**Table S2** Baseline Characteristics of NSTEMI

**Table S3** Baseline Characteristics of STEMI
