# Supplementary figures and images for: The association between anxiety disorders and in‐hospital outcomes in patients with myocardial infarction
Source: Clin Cardiol. 2020 Mar 18;43(6):622–9. doi: 10.1002/clc.23358 (PMC7298986; doi:10.1002/clc.23358)

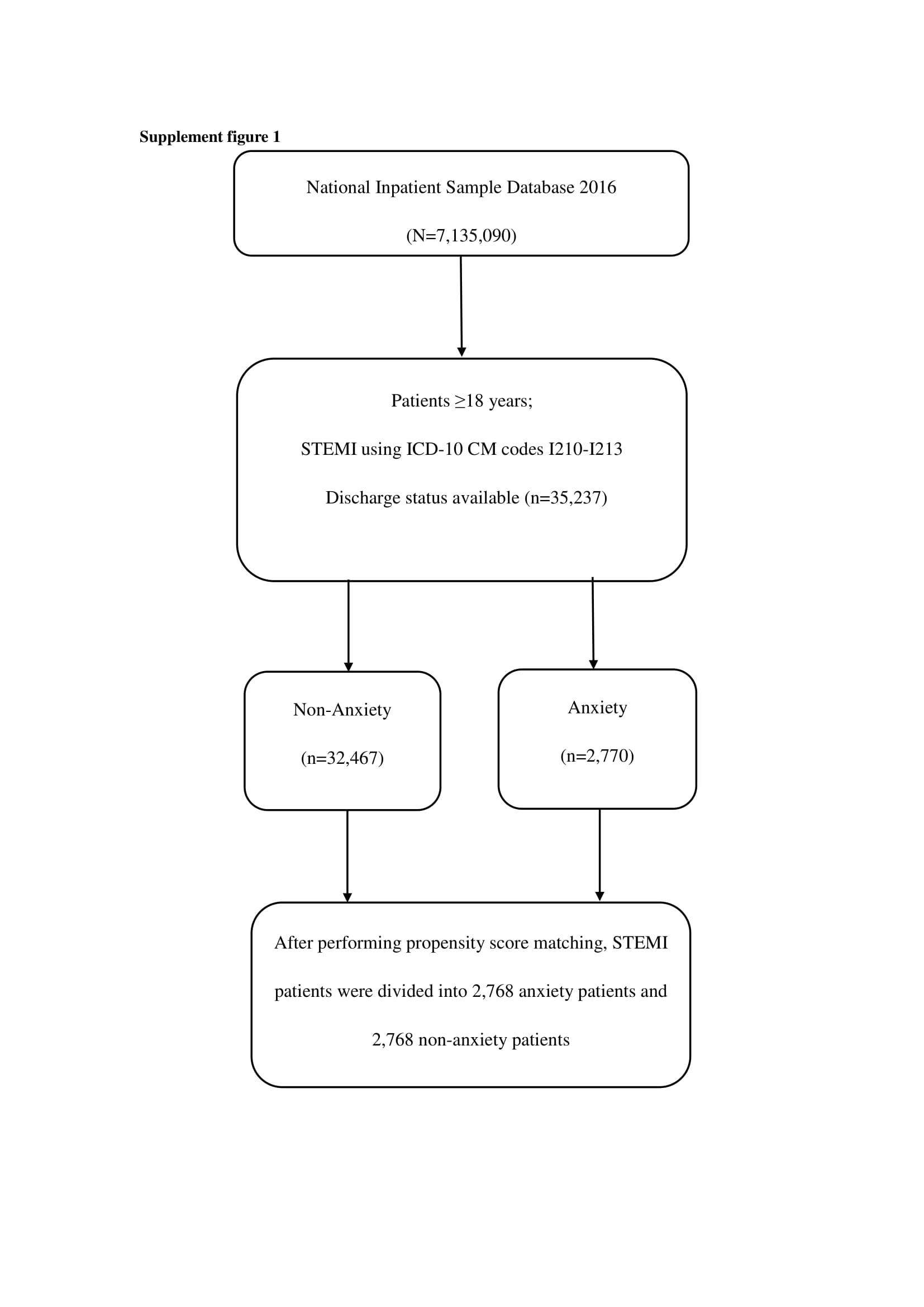

Supplement: Supplementary file 1 — Figure S1. Flow chart of the selection process for the final patient sample in the STEMI subgroup used in this study. Inclusion criteria were applied to the National Inpatient Sample 2016 database. All eligible patients were matched 1:1 based on propensity scoring to generate the anxiety vs nonanxiety comparison cohorts. ICD‐10‐CM code: Tenth Revision, Clinical Modification Code. STEMI: ST‐segment elevation myocardial infarction. [file CLC-43-622-s001.jpg]

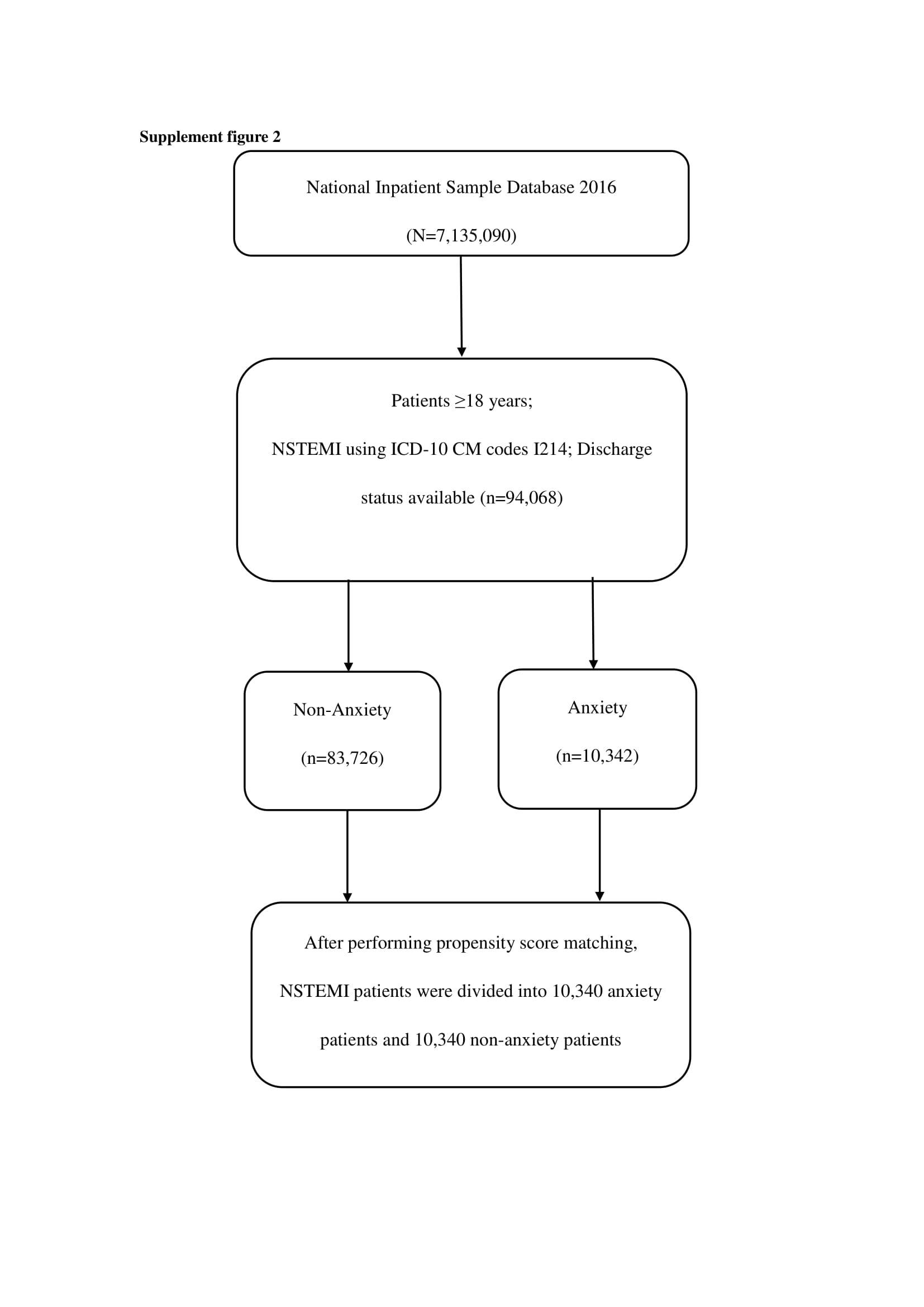

Supplement: Supplementary file 2 — Figure S2. Flow chart of the selection process for the final patient sample in the NSTEMI subgroup used in this study. Inclusion criteria were applied to the National Inpatient Sample 2016 database. All eligible patients were matched 1:1 based on propensity scoring to generate the anxiety vs nonanxiety comparison cohorts. ICD‐10‐CM code: Tenth Revision, Clinical Modification Code. NSTEMI: non‐ST elevation myocardial infarction. [file CLC-43-622-s002.jpg]
