# Supplementary material for: The association between anxiety disorders and in‐hospital outcomes in patients with myocardial infarction
Source: Clin Cardiol. 2020 Mar 18;43(6):622–9. doi: 10.1002/clc.23358 (PMC7298986; doi:10.1002/clc.23358)
Supplement: Supplementary file 3 — Table S1 International Classification of Disease, Version 10 (ICD‐10) Codes Used for Co‐Morbidity and Outcomes Identification [file CLC-43-622-s003.doc]

**Supplement Table 1** International Classification of Disease, Version 10 (ICD-10) Codes Used for Co-Morbidity and Outcomes Identification

| **Co-Morbidity/Outcomes** | **ICD-10 Code** |
| --- | --- |
| **MI** | I21, I210-I213, I214 |
| STEMI | I210-I213 |
| NSTEMI | I214 |
| **Anxiety Disorders** | F064, F40-F409, F41-F419 |
| **Comorbidities** |  |
| Smoking | F17-F17299, Z720, Z87891 |
| Hypertension | I10, I15-I159 |
| Diabetes | E10-E109, E11-E119, E13-E139 |
| Hyperlipidemia | E780-E785 |
| PAD | I70-I7092, I71-I719, I72-I729, I73-I739  I74-I479, I77-I779 |
| COPD | J41-J418, J42, J43-J439, J44-J449 |
| OSA | G473-G4739 |
| CKD | N18-N189 |
| Depressive disorders | F32-F329, F33-F339, F341 |
| History of MI | I252 |
| History of Stroke | I69- I69398, Z8673 |
| **Outcomes** |  |
| Cardiac arrest | I46-I469 |
| Cardiogenic shock | R570 |
| VA | I472, I490-I4902 |
| Acute CHF | I5021, I5023, I5031, I5034, I5041, I5043 |
| AKI | N17-N179 |
| ARF | J960-J9622 |

MI, myocardial infarction; STEMI, ST-segment elevation myocardial infarction; NSTEMI, non-ST elevation myocardial infarction; PAD, peripheral artery disease; COPD, chronic obstructive pulmonary diseases; OSA, obstructive sleep apnea; CKD, chronic kidney disease; VA, ventricular arrhythmia; CHF, congestive heart failure; AKI, acute kidney injury; ARF, acute respiratory failure
