# Supplementary material for: The association between anxiety disorders and in‐hospital outcomes in patients with myocardial infarction
Source: Clin Cardiol. 2020 Mar 18;43(6):622–9. doi: 10.1002/clc.23358 (PMC7298986; doi:10.1002/clc.23358)
Supplement: Supplementary file 4 — Table S2 Baseline Characteristics of NSTEMI [file CLC-43-622-s004.doc]

**Supplementary Table 2** Baseline Characteristics of NSTEMI

|  | **Unmatched Cohort** | |  |  | **Propensity-Matched Cohort** | | | |  | |
| --- | --- | --- | --- | --- | --- | --- | --- | --- | --- | --- |
| **Variables** | **NSTEMI without anxiety** | **NSTEMI with anxiety** | ***P* Value** | | |  | **NSTEMI without anxiety** | **NSTEMI with anxiety** | | ***P* Value** |
| **n** | 83726 | 10342 |  | | |  | 10340 | 10340 | |  |
| **Age, (mean (sd))** | 68.5 (13.5) | 66.4 (13.6) | <0.001 | | |  | 66.8 (13.4) | 66.4 (13.6) | | 0.02 |
| **Sex, n (%)** |  |  | <0.001 | | |  |  |  | | 0.095 |
| Male | 51524 (61.5) | 4339 (42.0) |  | | |  | 4477 (43.3) | 4339 (42.0) | |  |
| Female | 32160 (38.4) | 5997 (58.0) |  | | |  | 5860 (56.7) | 5995 (58.0) | |  |
| Unknown | 42 (0.1) | 6 (0.1) |  | | |  | 3 (0.0) | 6 (0.1) | |  |
| **Race, n (%)** |  |  | <0.001 | | |  |  |  | | <0.001 |
| White | 58214 (69.5) | 8195 (79.2) |  | | |  | 7847 (75.9) | 8193 (79.2) | |  |
| Black | 10262 (12.3) | 738 (7.1) |  | | |  | 1160 (11.2) | 738 (7.1) | |  |
| Hispanic | 6762 (8.1) | 674 (6.5) |  | | |  | 618 (6.0) | 674 (6.5) | |  |
| Asian/  Pacific Islander | 2294 (2.7) | 104 (1.0) |  | | |  | 162 (1.6) | 104 (1.0) | |  |
| Native American | 469 (0.6) | 39 (0.4) |  | | |  | 41 (0.4) | 39 (0.4) | |  |
| Other | 2303 (2.8) | 201 (1.9) |  | | |  | 149 (1.4) | 201 (1.9) | |  |
| Unknown | 3422 (4.1) | 391 (3.8) |  | | |  | 363 (3.5) | 391 (3.8) | |  |
| **Patient location, n (%)** |  |  | <0.001 | | |  |  |  | | 0.312 |
| "Central" counties of metro areas of >=1 million population | 21373 (25.5) | 2252 (21.8) |  | | |  | 2342 (22.6) | 2251 (21.8) | |  |
| "Finge" counties of metro areas of >=1 million population | 19494 (23.3) | 2465 (23.8) |  | | |  | 2474 (23.9) | 2464 (23.8) | |  |
| Counties in metro areas of 250,000-999,999 population | 16627 (19.9) | 2081 (20.1) |  | | |  | 2085 (20.2) | 2081 (20.1) | |  |
| Counties in metro areas of 50,000-249,999 population | 8553 (10.2) | 1219 (11.8) |  | | |  | 1138 (11.0) | 1219 (11.8) | |  |
| Micropolitan counties | 9615 (11.5) | 1276 (12.3) |  | | |  | 1281 (12.4) | 1276 (12.3) | |  |
| Non metropolitan or micropolitan counties | 7798 (9.3) | 1025 (9.9) |  | | |  | 986 (9.5) | 1025 (9.9) | |  |
| NA | 266 (0.3) | 24 (0.2) |  | | |  | 34 (0.3) | 24 (0.2) | |  |
| **Mean household income, n (%)** |  |  | 0.029 | | |  |  |  | | 0.782 |
| $1-$42,999 | 26032 (31.1) | 3319 (32.1) |  | | |  | 3245 (31.4) | 3318 (32.1) | |  |
| $43,000-$53,999 | 22143 (26.4) | 2732 (26.4) |  | | |  | 2763 (26.7) | 2732 (26.4) | |  |
| $54,000-$70,999 | 19229 (23.0) | 2385 (23.1) |  | | |  | 2388 (23.1) | 2385 (23.1) | |  |
| $71,000 or more | 14796 (17.7) | 1749 (16.9) |  | | |  | 1794 (17.4) | 1748 (16.9) | |  |
| Unknown | 1526 (1.8) | 157 (1.5) |  | | |  | 150 (1.5) | 157 (1.5) | |  |
| **Primary payer, n (%)** |  |  | <0.001 | | |  |  |  | | 0.291 |
| Medicare | 51875 (62.0) | 6376 (61.7) |  | | |  | 6440 (62.3) | 6375 (61.7) | |  |
| Medicaid | 7168 (8.6) | 1143 (11.1) |  | | |  | 1060 (10.3) | 1142 (11.0) | |  |
| Private including HMO | 18873 (22.5) | 2229 (21.6) |  | | |  | 2208 (21.4) | 2229 (21.6) | |  |
| Self-pay | 3127 (3.7) | 322 (3.1) |  | | |  | 358 (3.5) | 322 (3.1) | |  |
| No charge | 326 (0.4) | 27 (0.3) |  | | |  | 34 (0.3) | 27 (0.3) | |  |
| Other | 2272 (2.7) | 236 (2.3) |  | | |  | 235 (2.3) | 236 (2.3) | |  |
| Unknown | 85 (0.1) | 9 (0.1) |  | | |  | 5 (0.0) | 9 (0.1) | |  |
| **Hospital type, n (%)** |  |  | <0.001 | | |  |  |  | | 0.623 |
| Rural | 6754 (8.1) | 979 (9.5) |  | | |  | 939 (9.1) | 979 (9.5) | |  |
| Urban non-teaching | 23499 (28.1) | 2836 (27.4) |  | | |  | 2838 (27.4) | 2836 (27.4) | |  |
| Urban teaching | 53473 (63.9) | 6527 (63.1) |  | | |  | 6563 (63.5) | 6525 (63.1) | |  |
| **Hospital Region, n (%)** |  |  | <0.001 | | |  |  |  | | 0.1 |
| Northeast | 15350 (18.3) | 1941 (18.8) |  | | |  | 1960 (19.0) | 1940 (18.8) | |  |
| Midwest | 18598 (22.2) | 2522 (24.4) |  | | |  | 2519 (24.4) | 2521 (24.4) | |  |
| South | 34236 (40.9) | 4252 (41.1) |  | | |  | 4118 (39.8) | 4252 (41.1) | |  |
| West | 15542 (18.6) | 1627 (15.7) |  | | |  | 1743 (16.9) | 1627 (15.7) | |  |
| **Hospital Bed Size, n (%)** |  |  | 0.555 | | |  |  |  | | 0.17 |
| Small | 13888 (16.6) | 1675 (16.2) |  | | |  | 1665 (16.1) | 1675 (16.2) | |  |
| Medium | 25010 (29.9) | 3085 (29.8) |  | | |  | 2971 (28.7) | 3085 (29.8) | |  |
| Large | 44828 (53.5) | 5582 (54.0) |  | | |  | 5704 (55.2) | 5580 (54.0) | |  |
| **Comorbidities, n (%)** |  |  |  | | |  |  |  | |  |
| Smoking | 16873 (20.2) | 2802 (27.1) | <0.001 | | |  | 2684 (26.0) | 2800 (27.1) | | 0.07 |
| Hypertension | 44006 (52.6) | 5783 (55.9) | <0.001 | | |  | 5698 (55.1) | 5781 (55.9) | | 0.251 |
| DM | 35314 (42.2) | 3965 (38.3) | <0.001 | | |  | 4012 (38.8) | 3964 (38.3) | | 0.502 |
| Hyperlipidemia | 55157 (65.9) | 7050 (68.2) | <0.001 | | |  | 7063 (68.3) | 7048 (68.2) | | 0.834 |
| Obesity | 16092 (19.2) | 2228 (21.5) | <0.001 | | |  | 2240 (21.7) | 2227 (21.5) | | 0.839 |
| Depression | 5403 (6.5) | 3402 (32.9) | <0.001 | | |  | 3343 (32.3) | 3400 (32.9) | | 0.406 |
| History of MI | 13778 (16.5) | 1984 (19.2) | <0.001 | | |  | 1960 (19.0) | 1983 (19.2) | | 0.697 |
| OSA | 7812 (9.3) | 1250 (12.1) | <0.001 | | |  | 1228 (11.9) | 1249 (12.1) | | 0.668 |
| CKD | 23468 (28.0) | 2484 (24.0) | <0.001 | | |  | 2607 (25.2) | 2484 (24.0) | | 0.049 |
| History of Stroke | 9283 (11.1) | 1229 (11.9) | 0.016 | | |  | 1252 (12.1) | 1229 (11.9) | | 0.638 |
| PAD | 11769 (14.1) | 1486 (14.4) | 0.398 | | |  | 1464 (14.2) | 1486 (14.4) | | 0.676 |
| COPD | 16044 (19.2) | 2995 (29.0) | <0.001 | | |  | 2988 (28.9) | 2993 (28.9) | | 0.951 |

MI, myocardial infarction; STEMI, ST-segment elevation myocardial infarction; NSTEMI, non-ST elevation myocardial infarction; DM, diabetes mellitus; OSA, obstructive sleep apnea; CKD, chronic kidney disease; PAD, peripheral artery disease; COPD, chronic obstructive pulmonary disease
