# Supplementary material for: The association between anxiety disorders and in‐hospital outcomes in patients with myocardial infarction
Source: Clin Cardiol. 2020 Mar 18;43(6):622–9. doi: 10.1002/clc.23358 (PMC7298986; doi:10.1002/clc.23358)
Supplement: Supplementary file 5 — Table S3 Baseline Characteristics of STEMI [file CLC-43-622-s005.doc]

**Supplementary Table 3** Baseline Characteristics of STEMI

|  | **Unmatched Cohort** | |  |  | **Propensity-Matched Cohort** | | | |  | |
| --- | --- | --- | --- | --- | --- | --- | --- | --- | --- | --- |
| **Variables** | **STEMI without anxiety** | **STEMI with anxiety** | ***P* Value** | | |  | **STEMI without anxiety** | **STEMI with anxiety** | | ***P* Value** |
| **n** | 32467 | 2770 |  | | |  | 2768 | 2768 | |  |
| **Age, (mean (sd))** | 63.6 (13.2) | 62.1 (13.6) | <0.001 | | |  | 62.4 (13.4) | 62.2 (13.6) | | 0.439 |
| **Sex, n (%)** |  |  | <0.001 | | |  |  |  | | 0.362 |
| Male | 22839 (70.3) | 1410 (50.9) |  | | |  | 1463 (52.9) | 1410 (50.9) | |  |
| Female | 9615 (29.6) | 1359 (49.1) |  | | |  | 1304 (47.1) | 1357 (49.0) | |  |
| Unknown | 13 (0.0) | 1 (0.0) |  | | |  | 1 (0.0) | 1 (0.0) | |  |
| **Race, n (%)** |  |  | <0.001 | | |  |  |  | | <0.001 |
| White | 23246 (71.6) | 2254 (81.4) |  | | |  | 2164 (78.2) | 2252 (81.4) | |  |
| Black | 2799 (8.6) | 138 (5.0) |  | | |  | 263 (9.5) | 138 (5.0) | |  |
| Hispanic | 2529 (7.8) | 142 (5.1) |  | | |  | 145 (5.2) | 142 (5.1) | |  |
| Asian/  Pacific Islander | 921 (2.8) | 35 (1.3) |  | | |  | 40 (1.4) | 35 (1.3) | |  |
| Native American | 154 (0.5) | 11 (0.4) |  | | |  | 9 (0.3) | 11 (0.4) | |  |
| Other | 1058 (3.3) | 58 (2.1) |  | | |  | 33 (1.2) | 58 (2.1) | |  |
| Unknown | 1760 (5.4) | 132 (4.8) |  | | |  | 114 (4.1) | 132 (4.8) | |  |
| **Patient location, n (%)** |  |  | 0.009 | | |  |  |  | | 0.685 |
| "Central" counties of metro areas of >=1 million population | 8138 (25.1) | 626 (22.6) |  | | |  | 602 (21.7) | 625 (22.6) | |  |
| "Finge" counties of metro areas of >=1 million population | 7526 (23.2) | 666 (24.0) |  | | |  | 713 (25.8) | 665 (24.0) | |  |
| Counties in metro areas of 250,000-999,999 population | 7024 (21.6) | 619 (22.3) |  | | |  | 620 (22.4) | 619 (22.4) | |  |
| Counties in metro areas of 50,000-249,999 population | 3292 (10.1) | 322 (11.6) |  | | |  | 293 (10.6) | 322 (11.6) | |  |
| Micropolitan counties | 3545 (10.9) | 300 (10.8) |  | | |  | 312 (11.3) | 300 (10.8) | |  |
| Non metropolitan or micropolitan counties | 2789 (8.6) | 231 (8.3) |  | | |  | 223 (8.1) | 231 (8.3) | |  |
| NA | 153 (0.5) | 6 (0.2) |  | | |  | 5 (0.2) | 6 (0.2) | |  |
| **Mean household income, n (%)** |  |  | 0.599 | | |  |  |  | | 0.918 |
| $1-$42,999 | 9119 (28.1) | 769 (27.8) |  | | |  | 762 (27.5) | 769 (27.8) | |  |
| $43,000-$53,999 | 8594 (26.5) | 771 (27.8) |  | | |  | 760 (27.5) | 770 (27.8) | |  |
| $54,000-$70,999 | 7779 (24.0) | 650 (23.5) |  | | |  | 670 (24.2) | 650 (23.5) | |  |
| $71,000 or more | 6316 (19.5) | 529 (19.1) |  | | |  | 518 (18.7) | 528 (19.1) | |  |
| Unknown | 659 (2.0) | 51 (1.8) |  | | |  | 58 (2.1) | 51 (1.8) | |  |
| **Primary payer, n (%)** |  |  | <0.001 | | |  |  |  | | 0.189 |
| Medicare | 14511 (44.7) | 1321 (47.7) |  | | |  | 1326 (47.9) | 1320 (47.7) | |  |
| Medicaid | 3345 (10.3) | 341 (12.3) |  | | |  | 360 (13.0) | 340 (12.3) | |  |
| Private including HMO | 11210 (34.5) | 904 (32.6) |  | | |  | 867 (31.3) | 904 (32.7) | |  |
| Self-pay | 2122 (6.5) | 112 (4.0) |  | | |  | 143 (5.2) | 112 (4.0) | |  |
| No charge | 169 (0.5) | 10 (0.4) |  | | |  | 12 (0.4) | 10 (0.4) | |  |
| Other | 1061 (3.3) | 79 (2.9) |  | | |  | 58 (2.1) | 79 (2.9) | |  |
| Unknown | 49 (0.2) | 3 (0.1) |  | | |  | 2 (0.1) | 3 (0.1) | |  |
| **Hospital type, n (%)** |  |  | 0.002 | | |  |  |  | | 0.042 |
| Rural | 2051 (6.3) | 206 (7.4) |  | | |  | 179 (6.5) | 206 (7.4) | |  |
| Urban non-teaching | 9093 (28.0) | 703 (25.4) |  | | |  | 777 (28.1) | 702 (25.4) | |  |
| Urban teaching | 21323 (65.7) | 1861 (67.2) |  | | |  | 1812 (65.5) | 1860 (67.2) | |  |
| **Hospital Region, n (%)** |  |  | <0.001 | | |  |  |  | | 0.754 |
| Northeast | 5347 (16.5) | 532 (19.2) |  | | |  | 507 (18.3) | 532 (19.2) | |  |
| Midwest | 7399 (22.8) | 745 (26.9) |  | | |  | 736 (26.6) | 744 (26.9) | |  |
| South | 13237 (40.8) | 1033 (37.3) |  | | |  | 1067 (38.5) | 1033 (37.3) | |  |
| West | 6484 (20.0) | 460 (16.6) |  | | |  | 458 (16.5) | 459 (16.6) | |  |
| **Hospital Bed Size, n (%)** |  |  | 0.092 | | |  |  |  | | 0.553 |
| Small | 4439 (13.7) | 420 (15.2) |  | | |  | 391 (14.1) | 419 (15.1) | |  |
| Medium | 9461 (29.1) | 792 (28.6) |  | | |  | 793 (28.6) | 791 (28.6) | |  |
| Large | 18567 (57.2) | 1558 (56.2) |  | | |  | 1584 (57.2) | 1558 (56.3) | |  |
| **Comorbidities, n (%)** |  |  |  | | |  |  |  | |  |
| Smoking | 9799 (30.2) | 1051 (37.9) | <0.001 | | |  | 1047 (37.8) | 1049 (37.9) | | 0.978 |
| Hypertension | 18365 (56.6) | 1679 (60.6) | <0.001 | | |  | 1690 (61.1) | 1678 (60.6) | | 0.762 |
| DM | 9929 (30.6) | 803 (29.0) | 0.084 | | |  | 826 (29.8) | 803 (29.0) | | 0.516 |
| Hyperlipidemia | 19978 (61.5) | 1860 (67.1) | <0.001 | | |  | 1833 (66.2) | 1858 (67.1) | | 0.494 |
| Obesity | 5244 (16.2) | 531 (19.2) | <0.001 | | |  | 536 (19.4) | 531 (19.2) | | 0.892 |
| Depression | 1353 (4.2) | 812 (29.3) | <0.001 | | |  | 798 (28.8) | 810 (29.3) | | 0.745 |
| History of MI | 3628 (11.2) | 365 (13.2) | 0.002 | | |  | 348 (12.6) | 364 (13.2) | | 0.547 |
| OSA | 1976 (6.1) | 282 (10.2) | <0.001 | | |  | 283 (10.2) | 281 (10.2) | | 0.965 |
| CKD | 4110 (12.7) | 350 (12.6) | 0.995 | | |  | 359 (13.0) | 350 (12.6) | | 0.748 |
| History of Stroke | 2008 (6.2) | 216 (7.8) | 0.001 | | |  | 212 (7.7) | 216 (7.8) | | 0.88 |
| PAD | 2698 (8.3) | 259 (9.4) | 0.063 | | |  | 276 (10.0) | 259 (9.4) | | 0.467 |
| COPD | 3695 (11.4) | 480 (17.3) | <0.001 | | |  | 465 (16.8) | 480 (17.3) | | 0.617 |

MI, myocardial infarction; STEMI, ST-segment elevation myocardial infarction; NSTEMI, non-ST elevation myocardial infarction; DM, diabetes mellitus; OSA, obstructive sleep apnea; CKD, chronic kidney disease; PAD, peripheral artery disease; COPD, chronic obstructive pulmonary disease
